# Principles and applications of cell delivery systems for periodontal regeneration

**DOI:** 10.1111/j.1600-0757.2006.00156.x

**Published:** 2006

**Authors:** P. Mark Bartold, Yin Xiao, S. Petter Lyngstaadas, Michael L. Paine, Malcolm L. Snead

The management of periodontal defects has been an ongoing challenge in clinical periodontics. This is mainly a result of the fact that the tissues which comprise the periodontium, the periodontal ligament, and the cementum and alveolar bone, represent three unique tissues in their own right. Thus, reconstruction of the periodontium is not just a simple matter of regenerating one tissue but involves at least three quite diverse and unique tissues.

Resective surgical therapy, with or without osseous recontouring, was considered the norm during the 1950s and into the 1960s, in the belief that attainment of shallow pocket depths was a worthwhile goal. More recently, attention has been focused more on regenerative and reconstructive therapies, rather than on resective therapies. Currently, clinical and scientific research is focusing on a number of approaches for periodontal regeneration.

One approach requires the introduction of a ‘filler’ material into a periodontal defect, aiming to induce bone regeneration. Various types of bone grafts have been investigated to determine their ability to stimulate new bone formation. Of these, the following have been studied in detail:

- alloplastic materials, which are generally synthetic filler materials;
- autografts, which represent tissue grafted from one site to another in the same individual;
- allografts of tissue between individuals of the same species but with different genetic composition;
- xenografts, which consist of grafted materials between different species.

Although utilization of such grafting materials for periodontal defects may result in some gain in clinical attachment levels and radiographic evidence of bone fill, careful histologic assessment usually reveals that these materials have little osteoinductive capacity and generally become encased in a dense fibrous connective tissue (19).

In another approach to induce periodontal regeneration, polypeptide growth factors have been locally applied to the root surface in order to facilitate the cascade of wound-healing events that lead to the formation of new cementum and connective tissue. Among the myriad of growth factors currently characterized and available, platelet-derived growth factor and insulin-like growth factor-1 have been noted to enhance regeneration of periodontal defects in beagle dogs and monkeys (42, 61). Another promising group of polypeptide growth factors is the bone morphogenetic proteins, which offer good potential for stimulating bone and cementum regeneration (59). An extension of growth factor application to root surfaces is the application of cell-free matrix constructs to the root surface to aid cell repopulation and enhance regeneration. Enamel matrix proteins are such an example, and there is some evidence that these proteins can assist in the regeneration of periodontal tissues (24, 80). It is postulated that the enamel matrix derivative acts as a matrix enhancement factor, creating a positive environment for cell proliferation, differentiation and matrix synthesis (43, 23).

Yet another approach, known as guided-tissue regeneration, has been developed to achieve periodontal regeneration. This utilizes barrier membranes to guide and instruct the specialized cellular components of the periodontium to participate in the regenerative process. The guided-tissue regeneration concept was founded on sound scientific research and is based on the premise that the periodontal ligament contains all the progenitor cells required for the formation of bone, cementum and periodontal ligament (21, 35, 52). Periodontal regeneration can be induced through repopulation of the wound site by the progenitor cells. Although this procedure became widely accepted as a clinical procedure (35, 48), recent clinical evaluation has indicated that the clinical improvements obtained by this procedure are small and highly variable (10, 56, 78).

It now seems likely that a combination of several techniques may offer the best chance of a beneficial outcome. Through a combination of transplanted biomaterials containing appropriately selected and primed cells, together with an appropriate mix of regulatory factors and extracellular matrix components to allow growth and specialization of the cells, new therapies are emerging of significant clinical potential (4).

Tissue engineering is defined as the reconstruction of living tissues to be used for the replacement of damaged or lost tissue/organs of living organisms and is founded on the principles of cell biology, developmental biology and biomaterials science (49, 71, 60). This developing area of applied biomedical research is attracting considerable attention from both the private and government sectors because of its considerable economic and therapeutic potential (20, 44, 54). A clear distinction should be made between tissue engineering, which is the implantation of *in vitro*-seeded cells and matrices, and guided-tissue regeneration, which involves the use of acellular matrices that are spontaneously repopulated by the host after implantation.

Successful tissue engineering requires an interplay among three components (Fig. 1): the implanted and cultured cells that will create the new tissue; a biomaterial to act as a scaffold or matrix to hold the cells; and biological signaling molecules that instruct the cells to form the desired tissue type. This review will focus mainly on the use of scaffold materials used to transplant cells as a means of delivering either cells or proteins to a defect site.

## Periodontal tissue engineering

The principal requirements for tissue engineering are the incorporation of appropriate numbers of responsive progenitor cells and the presence of bioactive levels of regulatory signals within an appropriate extracellular matrix or carrier construct. Recent advances in mesenchymal stem cell isolation, growth factor biology and biodegradable polymer constructs have set the stage for successful tissue engineering of many tissues, of which the periodontium could be considered a prime candidate for such procedures. Preliminary studies have indicated that periodontal ligament and bone cells can be transplanted into periodontal sites with no adverse immunologic or inflammatory consequences (38, 46, 72, 82).

In order for successful periodontal regeneration to occur, it will be necessary to utilize and recruit progenitor cells that can differentiate into specialized cells with a regenerative capacity, followed by proliferation of these cells and synthesis of the specialized connective tissues which they are attempting to repair. Clearly, a tissue-engineering approach for periodontal regeneration will need to utilize the regenerative capacity of cells residing within the periodontium and would involve the isolation of such cells and their subsequent proliferation within a three-dimensional (3D) framework with implantation into the defect. The use of a prefabricated 3D scaffold, with the appropriate cells or instructive messages (e.g. growth factors and matrix-attachment factors) incorporated into it, may overcome many of the limitations associated with current regenerative technologies. With the success reported for other systems, a tissue-engineering approach to regenerate periodontal defects seems reasonable (4).

Despite the above positive outlook, there are still many issues that need to be dealt with before periodontal tissue engineering becomes commonplace. There are two main criteria for successful tissue engineering (5, 11, 64). First, there are the engineering principles, which relate to biomechanical properties of the scaffold, architectural geometry and space-maintaining properties. The second criterion relates to the biological functions of the engineered construct, including cell recruitment, cell proliferation, cell survival in culture and at the site of implantation, neovascularization and delivery of morphogenetic-, regulatory- and growth factors necessary for successful differentiation and tissue regeneration. In this review we will focus specifically on the design requirements of scaffolds for cell delivery and then discuss some of the materials and methods that have been used in recent years.

## Design requirements for cell seeding scaffolds

### Space maintenance within the defect site, and barrier or exclusionary functions

The important understanding that bone will grow into an adjacent tissue space, providing that space can be maintained and soft tissue ingrowth prevented, is not new (7, 30). These early observations, which led to the principles of guided-tissue regeneration, provide a fundamental concept when considering tissue engineering and placement of bioengineered matrices for regeneration. Thus, any engineered material should be of appropriate form and sufficient strength to allow placement into a defect that prevents subsequent collapse of the overlying tissues into the defect site. Indeed, the material should act in a manner consistent with the established principles of guided-tissue regeneration (62). These principles dictate that sufficient wound space and a suitable environment for regeneration will act synergistically to permit the uninhibited cascade of molecular and cellular events required for the regenerative process.

The necessary design features needed to obtain adequate space maintenance will include ease of handling and shaping, sufficient rigidity to withstand soft tissue collapse into the defect, and an internal structure compatible with cell attachment and colonization, as well as permitting the ingrowth of tissues compatible with those to be regenerated (11, 62, 79).

### Biocompatibility and design features

An ideal material for tissue-engineering scaffolds will require it to be either biocompatible with the tissues to be regenerated or biodegradable, allowing for gradual replacement by regenerated tissue (36). As both cell attachment and incorporation *in vitro*, as well as subsequent tissue maturation during *in situ* regeneration, are crucial features of tissue engineering, the amount of porosity and the pore size of the supporting 3D structure are important features that need to be taken into consideration when designing tissue-engineering scaffolds (9, 79). Moreover, biosafety of the tissue-engineered constructs also needs to be taken into consideration. Although no guidelines have yet been established for assessing the safety and efficacy of cell- and tissue-based tissue-engineered products, clearly these materials should be free from transmittable disease and immunologically inert, while not inducing an overexuberant inflammatory response (53). Indeed, the ability of the host to accept the implanted materials depends not only on the material used but also on the host reaction and the systemic health of the recipient (58).

### Incorporation of cells with an appropriate phenotype for ongoing periodontal regeneration

Bioengineered skin substitutes with incorporated cells and extracellular matrix have been available for some time (55). These artificial constructs can provide almost unlimited quantities of tissue for wound management and illustrate the potential of such an approach. With increasing knowledge of what constitutes cells with a ‘periodontal-regenerative phenotype’ (32), together with the identification of adult mesenchymal stem cells within the periodontal ligament (63), it should be possible to culture and subsequently incorporate these cells into a suitable biodegradable scaffold for immediate introduction into a periodontal defect.

More recently, viral vectors transduced into mesenchymal cells have been used as a novel means of introducing specific molecules to wound sites with the intention of stimulating tissue regeneration (51). Interestingly this technology has already been translocated into periodontal regeneration. Using a viral vector-delivery system, genetically altered cells that express certain growth factors necessary for periodontal regeneration (specifically platelet-derived growth factor) have been introduced into periodontal defects and appear to be able to significantly enhance the regenerative response in experimental animal models (1, 34). Such procedures introduce the problem of biosafety with regards to genetic manipulation and control over the steps, which will have to be dealt with prior to clinical acceptance.

For periodontal tissue engineering, potential sources of cells are from cementum (85), periodontal ligament (63) and bone (81, 82). It remains to be established whether the so-called progenitor cells that reside in these tissues can be isolated and propagated in culture for future seeding (86).

### Incorporation and bioavailability of instructive messages

Growth and differentiation factors are essential ingredients for tissue regeneration. Hence, the synthetic scaffold used for tissue engineering should not only be bioresorbable but also constructed from a material with a suitable affinity for the adsorption of appropriate growth/differentiation factors as well as integrins, cell receptors and other instructive molecules normally found in regenerating tissues (8, 28, 65). Notwithstanding this important requisite, choosing the ‘correct’ agent or agents is a formidable task. The plethora of bioactive molecules involved in tissue regeneration will make the rational selection of specific agents very difficult. However, as our understanding of the signaling molecules required for optimal growth, differentiation and gene expression becomes clearer, it is anticipated that these agents may be incorporated into engineered matrices for regenerative purposes based on sound biologic principles.

Although biological molecules can relatively easily be conjugated to an artificial tissue-engineered scaffold, issues relating to suitable release and delivery kinetics will become the major focus of interest. Obstacles yet to be overcome in this regard include controlling the concentration, local duration and spatial distribution of these bound factors. Indeed, the control and containment of the agent are paramount for its effectiveness and safety (8).

Recently, bioengineers have devised novel methods to create a self-assembled molecular structure that responds to ultrasonic energy by releasing a burst of entrapped drug (37). Moreover, the self-assembling structure is a barrier to drug release in the absence of ultrasonic energy, reducing the problem of overt leakage from an indwelling device. The prototype for this has shown favorable release rates for insulin, as well as for an antibiotic compound, ciprofloxacin, when triggered by ultrasonic energy. In these tests, essentially no drug leakage occurred in the absence of an appropriate energy signature. This work also suggests that self-assembling structures could be devised to serve as a barrier while simultaneously serving as an ultrasonic responsive drug-release device to promote tissue-regeneration strategies. Such molecularly triggered devices might also permit the placement of an appropriate therapeutic regimen that would be released only by the practitioner when required to match a clinical scenario.

## Regulating cell activity through scaffold design

It has been recognized, for many years, that the microenvironment in which a cell resides dictates many functions and phenotypes (27). Thus, it seems logical that the construction and design of a cell-seeding scaffold must take into account microenvironment design features to induce the appropriate gene expression in cells forming new tissues. The control of gene expression by cells within a scaffold can be regulated via interactions with the adhesion surface, with other cells in the vicinity or, as described above, incorporated growth and differentiation factors in the scaffold. Accordingly, cell-seeding scaffolds must provide the correct combination of these factors, according to the tissues to be regenerated, if one is to achieve successful gene expression and tissue regeneration. To date, little work has been carried out in this complex area, although early studies have begun to utilize specific cell-attachment peptide sequences (the RGD sequence for integrins), pore size and surface texture in attempts to improve tissue integration and regeneration.

When considering scaffold design, many tissues depend upon mechanical stimuli to regulate gene expression and thus tissue composition. The most obvious example of this is bone and tendon, although it is likely that the periodontal ligament should also be considered in this context. In order to engineer such functional tissues, the correct mechanical stimuli will need to be conveyed to the developing tissues within the cell/scaffold construct. To date, because of the complexities of such systems, very few studies have addressed these issues (41).

## Types of cell-delivery devices and scaffolds

A common approach to tissue engineering is to use an exogenous 3D extracellular matrix to engineer new tissues using isolated cells. The exogenous matrix constructs are designed to encourage cells to come into contact with it in a suitable 3D environment and to provide structural support for the newly forming tissues. More recently, a variant of this approach has been used to isolate cells from biopsy specimens and expand them *in vitro* prior to seeding onto a suitable 3D matrix. In doing so, the cells are allowed to either develop into a new tissue *in vitro* or are immediately transplanted to a particular site to create new functional tissue, which is integrated within the recipient site.

Most cell-seeding scaffolds are fabricated from two classes of biomaterials, derived from either synthetic or natural products. In addition, they may be constructed from either resorbable or nonresorbable materials (Table 1). Natural products (e.g. collagen) are known to have specific desirable biologic properties, such as permitting cell interactions, but have the disadvantages of being derived from animal or human tissue, leading to issues regarding availability, safety and batch-to-batch variations. In contrast, synthetic materials can be produced on a large scale, to specific design criteria and from generally inert, biocompatible and biodegradable materials. Not surprisingly, there has been a plethora of materials developed and studied over the years, each claiming specific and unique advantages over ‘competitor’ products. Therefore, the following discussion is restricted to examples of cell carrier and delivery devices currently under investigation and of relevance to periodontal regeneration.

## Nonresorbable materials

### Expanded polytetrafluoroethylene (ePTFE, Goretex^™^)

**Fig. 2.**
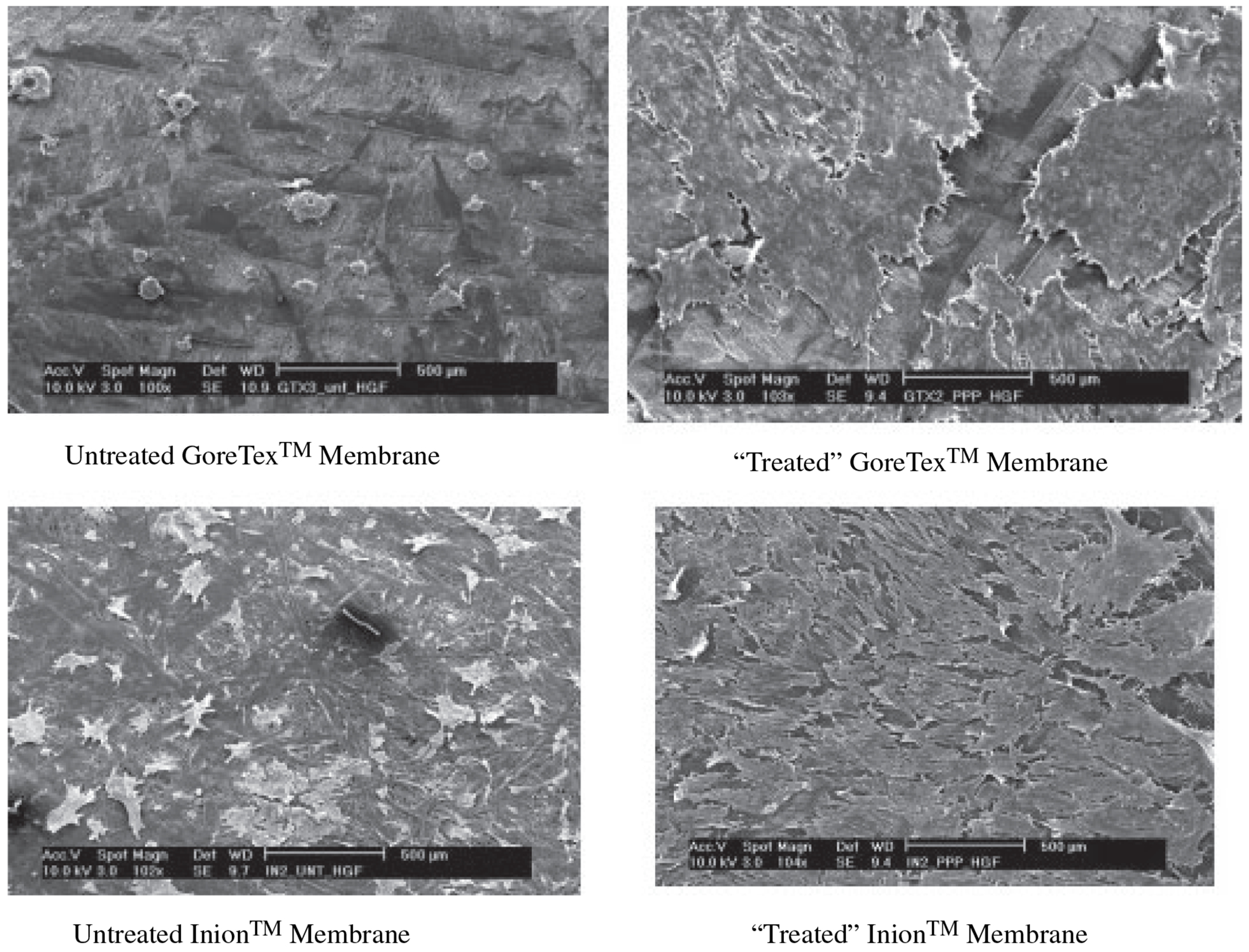
Cell attachment to guided tissue regeneration membranes as cell delivery devices.

Membranes made from expanded polytetrafluoroethylene have traditionally been used as guided-tissue barrier membranes. However, it is possible that these membranes could also be used to nurture specific cells that are expanded *ex vivo* and then delivered to a defect site. In the same context, almost any guided-tissue regeneration membrane could be used in such a manner, utilizing either nonresorbable or resorbable materials (Fig. 2).

### Porous ceramic scaffolds

Several porous ceramic scaffolds have been examined for their utilization as cell-delivery materials. In general, many of these materials have been developed and investigated with regard to bone tissue engineering (69). For these purposes, the ideal scaffold should be a porous material with good biocompatibility and possess osseointegrative capabilities, high mechanical strength and biodegradability. Some ceramic materials have the former two properties but, to date, no porous scaffolds satisfy both of the latter two properties.

Hydroxyapatite is an example of a material with good mechanical properties but, owing to its porosity, poor strength. Another problem with porous hydroxyapatite is the lack of interconnectivity of the pores, making neovascularization of any implant almost impossible. Many studies have shown that hydroxyapatite scaffolds cultured with bone cells have good osteogenic potential (16).

Biodegradable porous ceramic materials have also been developed and investigated. Of these, the most popular material possessing high biocompatibility and biodegradability is beta-tricalcium phosphate. When implanted alone at extraskeletal sites, beta-tricalcium phosphate undergoes rapid degradation with little bone formation. Owing to this rapid degradation of beta-tricalcium phosphate and its associated poor mechanical properties, research has focused on mixed calcium phosphates, such as mixtures of beta-tricalcium phosphate and hydroxyapatite or beta-tricalcium phosphate and polymers. These hybrid materials appear to be reliable vehicles for cell delivery, with studies showing good tissue formation associated with the implanted cells (16, 22).

### Titanium mesh

Another nonresorbable scaffold that has received considerable attention in recent years is titanium mesh (33). This material has good mechanical properties regarding stiffness and elasticity and is relatively easy to handle during surgical placement. The lack of bioresorbabilty of this material can be beneficial for the management of large osseous defects whereby the mesh retains sufficient rigidity to avoid collapse, which would be expected of teflon membranes or biodegradable scaffolds. Various studies have indicated that this material is suitable for supporting the growth and osteogenic expression of bone marrow cells (73, 75, 77). Through various surface treatments, including the addition of fibronectin, collagen or calcium phosphate, the rate and amount of bone formation by implanted cells into titanium mesh scaffolds can be regulated (74, 76).

## Resorbable materials

Resorbable materials offer the significant advantage that they do not need to be retrieved at a later date from the site of implantation. They include materials such as polyesters of naturally occurring alpha-hydroxy acids, amino acid-based polymers, alginate, and natural materials such as collagen and reconstituted extracellular matrix proteins.

### Alpha-hydroxy acids

The alpha-hydroxy acid polymers include polyglycolic acid, poly(l-lactic acid) and copolymers of poly(lactic-*co*-glycolic acid). These materials have been used extensively for cell seeding in tissue engineering (66, 83). Their ester bonds are rather susceptible to hydrolysis and thus degrade by nonenzymatic means. Accordingly, these natural breakdown products are removed from the site of implantation by normal tissue respiratory routes and do not generally elicit a foreign body response resulting in massive macrophage infiltration and chronic inflammation. Through specific chemical manipulation, these materials can be fabricated to degrade over long or short periods of time, depending on the clinical need. These materials can also be easily manufactured into preformed sizes and shapes, as dictated by the site of the defect and its anatomy.

However, these materials are hydrophobic and are processed under quite stringent (biologically adverse) conditions, which usually makes factor incorporation and attachment or entrapment of cells difficult. Recently, a biodegradable copolymer of l-lactic acid [d-lactic acid, glycolic acid and trimethylene carbonate (Inion Ltd, Tampere, Finland)] has been developed. Although originally developed for use in dentistry as exclusionary barrier membranes, these biodegradable membranes offer good potential as cell-delivery devices, with the advantage that they seem to allow cell attachment more readily than other inert materials, such as expanded polytetrafluoroethylene (Fig. 2).

### Alginate

**Fig. 3.**
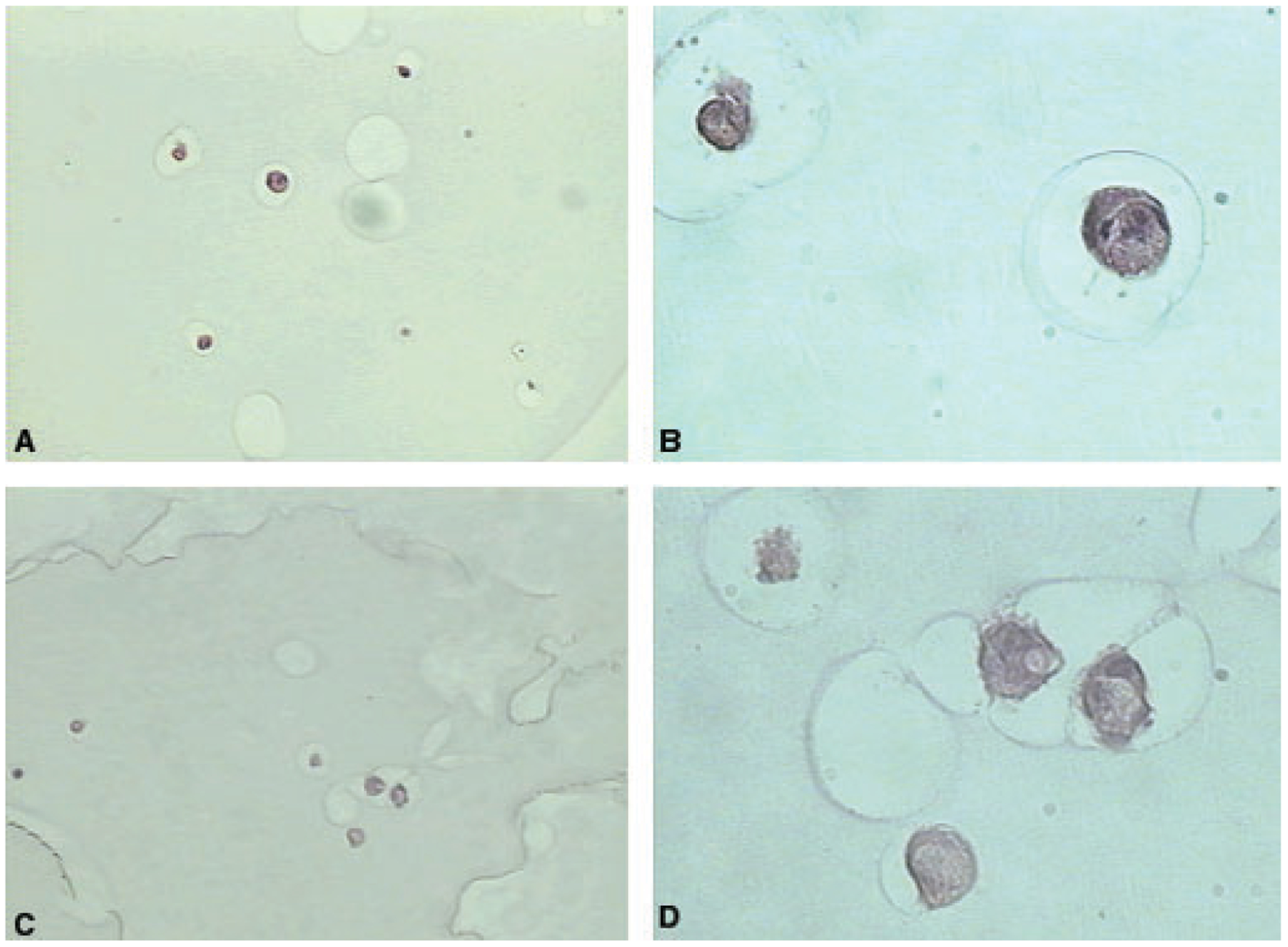
Culture of human periodontal ligament cells in alginate beads. (A) Cells cultured in alginate beads for 2 days – 10×. (B) Cells cultured in alginate beads for 2 days – 40×. (C) Cells cultured in alginate beads for 8 days – 20×. D. Cells cultured in alginate beads for 8 days – 40×.

An alternative to alginate gels as a cell carrier is the incorporation of cells into beads of alginate (Fig. 3) (70). The technique is based on entrapment of individual cells and tissues into an alginate droplet that is transformed into a rigid bead by gelation in a divalent cation-rich solution. The cells are surrounded by a nondegradable, selectively permeable barrier, which isolates the transplanted cells from host tissue and larger molecular weight solutes. Such implants are considered immunoprotective as they prevent immune cells and soluble complexes from killing the transplanted cells, and this property negates the need for immunosuppressant use (68). While these systems may be used to deliver cells to a specific site, because of the entrapment of the cells within an encapsulated environment, there is little opportunity for direct and immediate cell–matrix interaction at the site of implantation. Moreover, as a result of the semipermeable nature of the beads, the soluble factors made by the entrapped cells can be released at the implantation site to guide regenerated tissues. In recent years, these devices have been more appropriately developed as drug-delivery devices than cell-delivery devices for tissue engineering (68).

### Amino acid polymers

Amino acid-based polymers have also been used as scaffolds for cell seeding. These scaffolds can be synthesized using fermentation and gene transfer technology to produce molecules that resemble natural amino acid-containing matrix molecules, such as collagens, and elastin (29). While these materials have the advantage of being able to interact well with cells, issues of biosafety (immunogenicity), large-scale production and purification from unwanted contaminants remain a problem (36).

### Scaffolds derived from natural products

A variety of materials derived from natural products have been investigated as cell-seeding scaffold materials. Cross-links between polymer chains and various chemical bonds are often used to confer structural integrity to these products. Such materials are produced under relatively mild conditions and possess structural and mechanical properties reminiscent of the extracellular matrix, which can act as space fillers, bioactive molecule delivery devices or cell scaffolds. Examples of such materials are given in Table 1 and include both synthetic and naturally derived polymers. Of these, naturally derived polymers, such as alginate, collagen, chitosan and hyaluronate, have been extensively studied as cell-delivery vehicles. These materials provide an excellent means to transplant cells and form 3D cell-filled matrices. Nonetheless, such materials have several problems, including variability in composition, poor mechanical properties and degradation rates that are time-limited and difficult to control.

Hyaluronate has considerable potential as an optimal biomaterial for tissue engineering, given the significant role it plays during organogenesis, cell migration and development in general (67). Modifications to hyaluronan include esterification and cross-linking to provide some structure and rigidity to the gel for cell-seeding purposes. These biopolymers are immunologically inert and completely biodegradable (6, 14) and support the growth of fibroblasts, chondrocytes and mesenchymal stem cells (12, 57, 84).

Chitosan, a biopolymer that is structurally very similar to naturally occurring glycosaminoglycans and is biodegradable in mammals, has been used quite extensively as a tissue-engineering scaffold. While chitosan can support cell attachment for cell-delivery purposes (3, 18), it is not strongly supportive of cell growth (50). Accordingly, chitosan needs to be either modified chemically or conjugated with other molecules or peptides to enhance its biocompatibility for cell attachment (40, 45).

**Fig. 4.**
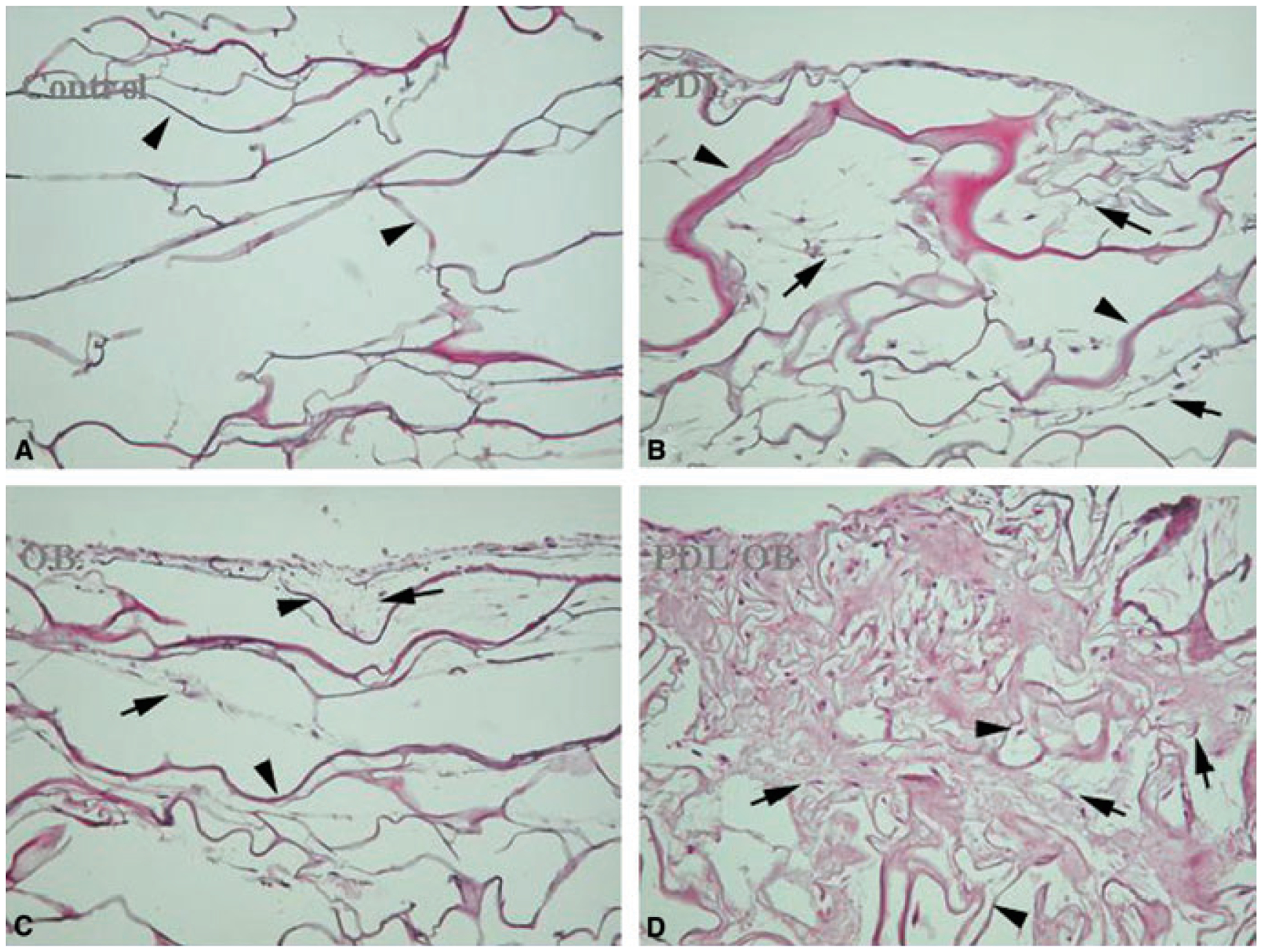
Culture of human periodontal ligament fibroblasts and osteoblasts as single or mixed co-cultures in a collagen sponge. (A) Control – no cells. (B) Periodontal ligament fibroblasts. (C) Osteoblasts. (D) Coculture of osteoblasts and periodontal ligament fibroblasts.

**Fig. 5.**
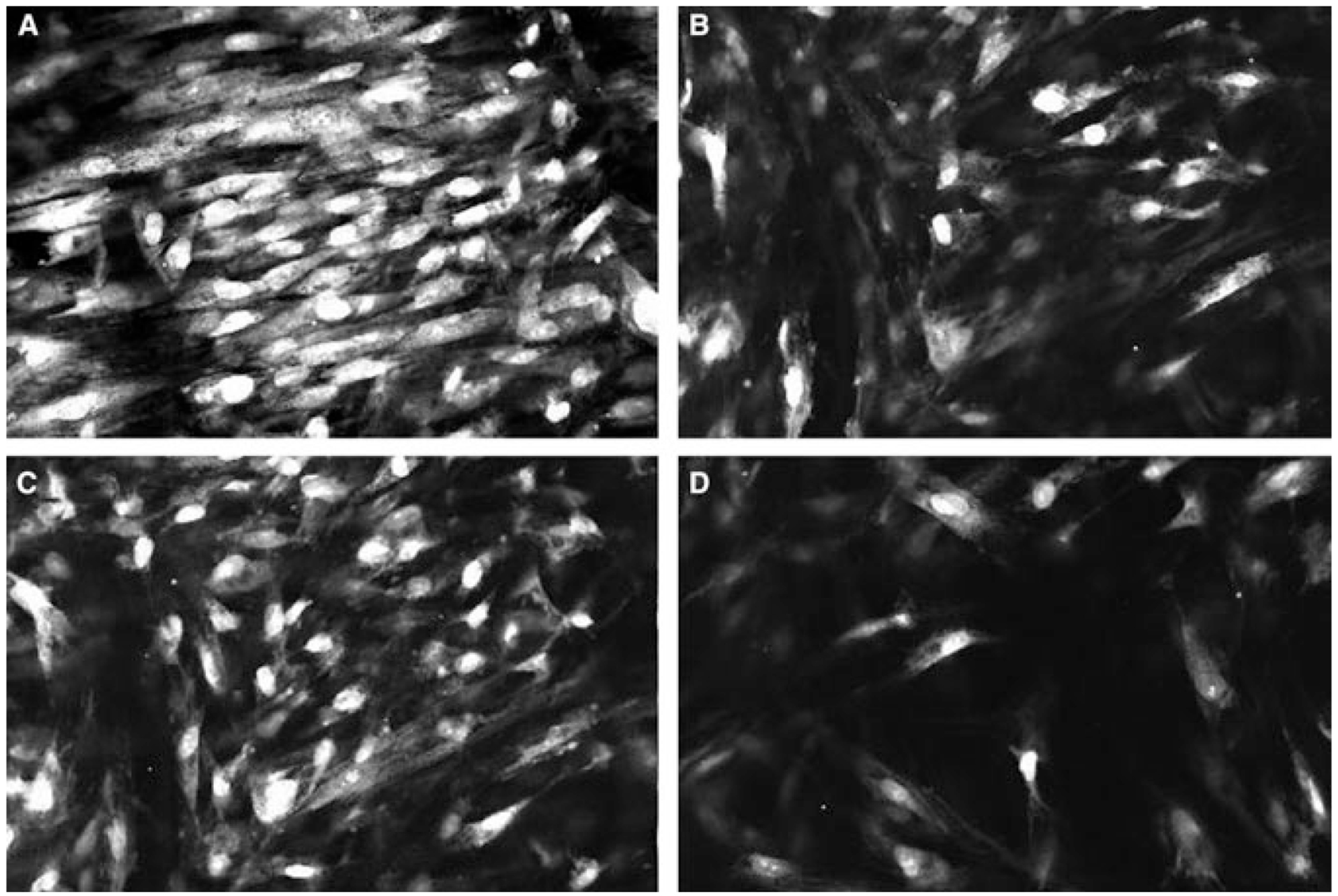
Confocal microscopy of cell penetration into collagen scaffold. (A) Cells on the surface of collagen sponges. (B) Cells in collagen sponges at 20 μm from surface. (C) Cells in collagen sponges at 60 μm from surface. (D) Cells in collagen sponges at 100 μm from surface.

Collagen scaffolds have been investigated as a cell-delivery device for many years (39). Collagen is regarded as one of the most useful biomaterials owing to its excellent biocompatibility and safety associated with its biological characteristics, such as biodegradability and weak antigenicity (Fig. 4). In this regard, collagen has been used for tissue engineering purposes, including skin replacement, bone substitutes, artificial blood vessels and valves. In the context of this review, collagen sponges and membranes offer particular features for cell integration and tissue engineering (Fig. 5). Cells can readily be seeded into collagen sponges or membranes, cultured and then introduced into a tissue defect site, where they can effect tissue repair and regeneration (81).

### Synthetic hydrogels

Synthetic hydrogels, such as poly(ethylene glycol) and poly(ethylene oxide), are also showing considerable promise for use as a 3D scaffold for cell delivery. By varying the initial cross-linking density, the degradation profiles of the gel can be controlled (13). In addition, it is possible to construct thermally reversible hydrogels as well as gels which can be degraded by either hydrolytic or enzymatic means (2, 47). Poly(ethylene oxide) is currently approved by the U.S. Food and Drug Administration for several applications in medicine and, together with polyglycolic acid, is one of the most common synthetic materials used for tissue engineering (17).

### Extracellular matrix scaffolds

Extracellular matrix extracts or derivatives have been developed as commercial products for cell delivery. In particular, many skin and extracellular matrix substitutes, such as Matrigel^™^ (BD Biosciences, San Jose, CA), Dermagraft^™^ (Advanced Tissue Sciences Inc, La Jolla, CA), Apligraf^™^ (Organogenesis Inc., Canton, MA) and Epidex^™^ (Modex Therapeutiques SA, Lausanne, Switzerland), have been developed to allow the incorporation of *ex vivo*-expanded cells. Nonetheless, these products, as well as other acellular therapies, such as PV702 (GroPep Pty, Ltd, Adelaide, South Australia) and Allograft^™^ (Life Cell Corp, Branchburg, NJ), incorporate animal-derived products and/or allogenic tissues and thus constitute a potential source of pathogens. Consequently, they are unlikely to be routinely used as cell delivery devices in the longer term.

**Fig. 6.**
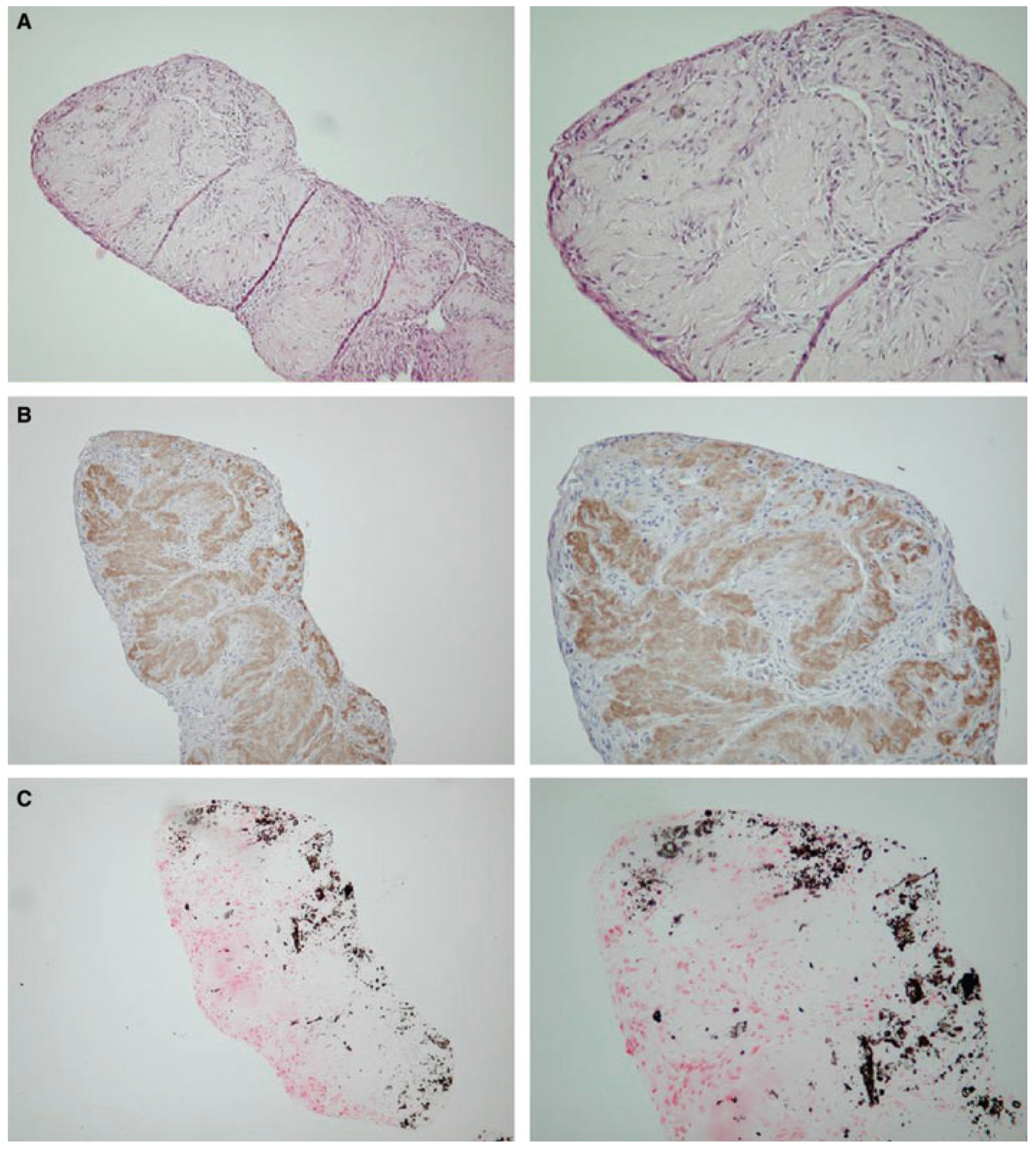
(A) Long-term culture of human osteoblasts to produce *in vitro* cell/matrix complex. Left: low power. Right: high power. (B) Type 1 collagen distribution in long-term *in vitro* produced cell/matrix. Left: low power. Right: high power. (C) Von Kossa staining for calcium deposition in long-term *in vitro* produced cell/matrix. Left: low power. Right: high power. Reproduced with permission from Xiao et al. (82).

*In vitro*-produced extracellular matrix also offers potential as a biodegradable scaffold for cell delivery. The use of extracellular matrix materials as scaffolds for the repair and regeneration of tissues is receiving increased attention. In a recent study we have shown that extracellular matrix formed by osteoblasts *in vitro* can be used as a scaffold for osteoblast transplantation and induce new bone formation in critical-size osseous defects *in vivo* (82). Human osteoblasts were cultured for 3 weeks to produce their own structured extracellular matrix (Fig. 6). The cells and self-produced matrix was then implanted into critical-size osseous defects. The cells inside the matrix survive and proliferate at the recipient sites. It was found that bone-forming cells differentiated from both transplanted human osteoblasts and activated endogenous mesenchymal cells.

## New directions

The field of tissue-engineering constructs and scaffolds is expanding at a very rapid rate. It would, within the confines of this review, be impossible to detail all of the most recent developments. However, there are two particular new directions in which the authors are specifically interested that involve the co-culture of cells and nanotechnology.

In attempts to deliver cells to a complex environment, such as the periodontium, it is possible that delivery of cells of multiple phenotypes may be required. For example, if one wanted to regenerate both periodontal ligament and alveolar bone, the possibility exists of bilaterally seeding periodontal ligament cells on one side of a bioscaffold and osteoblasts on the opposite side. While preliminary studies have begun to address such an approach, little definitive data are yet available. In a similar vein, it is not too difficult to envisage the engineering of a periodontal ligament-like matrix from periodontal ligament fibroblasts to which one side would then be seeded with cementoblasts and the opposite side seeded with osteoblasts. Using such an approach it may be possible to fully reconstitute various compartments of the periodontium *in vitro* and then implant such constructs into periodontal defects.

Advances in nanotechnology will also undoubtedly allow the synthesis of materials with desirable nanoscale structures. Nanotechnology is the science of engineering at the individual molecular level to produce materials of hitherto unthought of properties. Already, self-assembly systems have been described and fabricated which mimic many features of the extracellular matrix. For example, nanostructured fibrous scaffold, reminiscent of extracellular matrix, can be constructed using the pH-induced self-assembly of a peptide amphiphile. After cross-linking, the fibers are able to direct mineralization of hydroxyapatite to form a composite material in which the crystallographic c-axes of hydroxyapatite crystals are aligned with the long axes of the collagen fibrils. This alignment is the same as that observed between collagen fibrils and hydroxyapatite crystals in bone (25). Similarly, self-assembling biomaterials, with molecular features designed to interact with cells and scaffolds for tissue regeneration, have been reported (31). These nanofibers display attachment domains, in the form of RGD motifs, that are incorporated into the amphiphiles that self-assemble into nanofibers. The density of the RGD motif, and perhaps soon, alternative cell-signaling motifs, can be incorporated into the amphiphile for creation of a nanofiber with unique surface properties. Cells can be embedded in the nanofiber to resemble a ‘native’ extracellular matrix (26). As these materials are chemically synthesized, they present no risk of viral contaminants, as might occur for natural compounds recovered from biological sources, such as pigs or humans.

## Concluding comments

The study of scaffold materials for use in tissue engineering should lead to improved predictability of this new technology based on cell and molecular biology. In the future it will become increasingly important to consider the concepts of scaffolds that are not only space making and exclusionary, but also biocompatible and able to elicit appropriate gene expression by the cells for which it is providing the carrier capacity. Understanding the complex design features necessary for successful tissue engineering will help this technique to become an accepted biomedical procedure.

## Figures and Tables

**Fig. 1.**
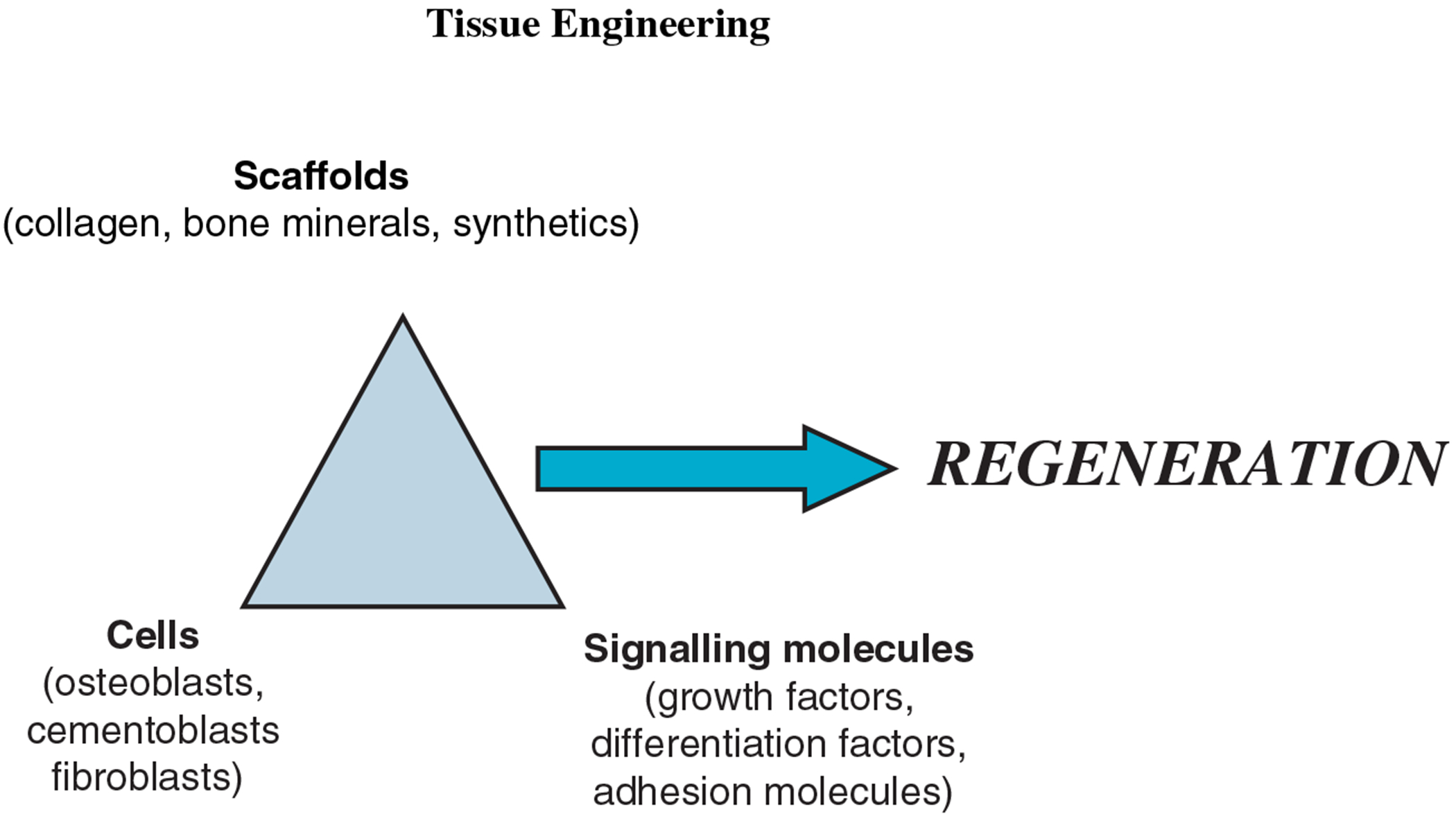
Tissue engineering determinants in periodontics.

**Table 1.** Examples of cell-delivery devices and scaffolds in periodontics

**Nonresorbable**
Expanded polytetrafluoroethylene (ePTFE)
Ceramic
Titanium mesh
**Resorbable**
Alpha-hydroxyacids
Polyglycolic acid
Poly(l-lactic acid)
Copolymers of poly(lactic-*co*-glycolic acid)
Amino acid-based polymers
Collagen-like proteins
Elastin-like proteins
Natural products
Collagen
Hyaluronan
Chitosan
Gelatin
Fibrin
Alginate
Synthetic hydrogels
Poly (ethylene glycol)
Poly (ethylene oxide)
Matrix extracts
Matrigel
